# Adaptation of Saccadic Sequences with and without Remapping

**DOI:** 10.3389/fnhum.2016.00359

**Published:** 2016-07-22

**Authors:** Delphine Lévy-Bencheton, Aarlenne Zein Khan, Denis Pélisson, Caroline Tilikete, Laure Pisella

**Affiliations:** ^1^Centre de Recherche en Neurosciences de Lyon (CRNL), ImpAct team, Inserm U1028, CNRS UMR 5292, Lyon1 UniversityBron, France; ^2^School of Optometry, University of MontrealMontreal, QC, Canada

**Keywords:** saccade adaptation, updating, remapping, backward, forward, double-step saccades, credit assignment

## Abstract

It is relatively easy to adapt visually-guided saccades because the visual vector and the saccade vector match. The retinal error at the saccade landing position is compared to the prediction error, based on target location and efference copy. If these errors do not match, planning processes at the level(s) of the visual and/or motor vector processing are assumed to be inaccurate and the saccadic response is adjusted. In the case of a sequence of two saccades, the final error can be attributed to the last saccade vector or to the entire saccadic displacement. Here, we asked whether and how adaptation can occur in the case of remapped saccades, such as during the classic double-step saccade paradigm, where the visual and motor vectors of the second saccade do not coincide and so the attribution of error is ambiguous. Participants performed saccades sequences to two targets briefly presented prior to first saccade onset. The second saccade target was either briefly re-illuminated (sequential visually-guided task) or not (remapping task) upon first saccade offset. To drive adaptation, the second target was presented at a displaced location (backward or forward jump condition or control—no jump) at the end of the second saccade. Pre- and post-adaptation trials were identical, without the re-appearance of the target after the second saccade. For the 1st saccade endpoints, there was no change as a function of adaptation. For the 2nd saccade, there was a similar increase in gain in the forward jump condition (52% and 61% of target jump) in the two tasks, whereas the gain decrease in the backward condition was much smaller for the remapping task than for the sequential visually-guided task (41% vs. 94%). In other words, the absolute gain change was similar between backward and forward adaptation for remapped saccades. In conclusion, we show that remapped saccades can be adapted, suggesting that the error is attributed to the visuo-motor transformation of the remapped visual vector. The mechanisms by which adaptation takes place for remapped saccades may be similar to those of forward sequential visually-guided saccades, unlike those involved in adaptation for backward sequential visually-guided saccades.

## Introduction

Saccades are brief and fast eye movements that we use to sample our visual environment. We make 4–5 saccades every second toward peripheral visual targets and the accuracy of these saccades is maintained despite neural noise, bias and fatigue due to adaptation mechanisms. In laboratory conditions, saccades planning and adaptation mechanisms are mostly studied using relatively simple experimental designs such as the classic saccadic adaptation paradigm where a peripherally presented target is systematically shifted during the saccade to it (McLaughlin, 1967; Hopp and Fuchs, 2004). Over a very short time-scale, plasticity mechanisms induce an enduring change of saccade amplitude when the target is shifted backward or forward during the saccade (Hopp and Fuchs, 2004; Pélisson et al., 2010). A multitude of studies have shown convincing evidence that saccadic adaptation is driven by the predicted error rather than the retinal post-saccadic error (Bahcall and Kowler, 2000; Wong and Shelhamer, 2011; Collins and Wallman, 2012; Herman et al., 2013). This is supported by studies showing that participants rarely adapt fully to a target jump, particularly in forward adaptation conditions and that saccades will continue to adapt even when the retinal error is manipulated to be zero by placing the target at the fovea after the saccade (Henson, 1978; Robinson et al., 2003; Havermann and Lappe, 2010). Thus, it is suggested that the saccadic system maintains a certain amount of undershoot to targets, and therefore maintains a certain amount of predicted error by planning saccades that do not fully compensate for the eye-target distance. Adaptation is thought to occur only when the observed error at the saccade landing position does not equal the predicted error. Moreover, it has been suggested that this drive to maintain a certain amount of undershoot is also responsible for the difference in adaptation between backward and forward target jumps; backward target jumps are more likely to lead to overshoots leading to greater adaptation compared to forward target jumps which simply lead to larger undershoots which are more acceptable. The calculation of this predicted error is relatively simple for single targets presented in the periphery. Because the target was viewed peripherally, its localization is relatively accurate but slightly under-estimated. This leads to a relatively accurate but hypometric motor vector, resulting in relatively small variability of saccade landing positions for peripherally viewed targets with a saccadic gain below one. Indeed, it is a classical observation that gain of visually-guided saccades tends to be less than one (0.9–0.95), meaning that by definition, the saccade error increases as the eccentricity of the target increases (Becker, 1989). Thus the saccadic system can trust that the discrepancy between the predicted error and the actual error is an error in the saccade planning process. This can then be quickly modified until the predicted error is equal to the actual error.

**Figure 1:**
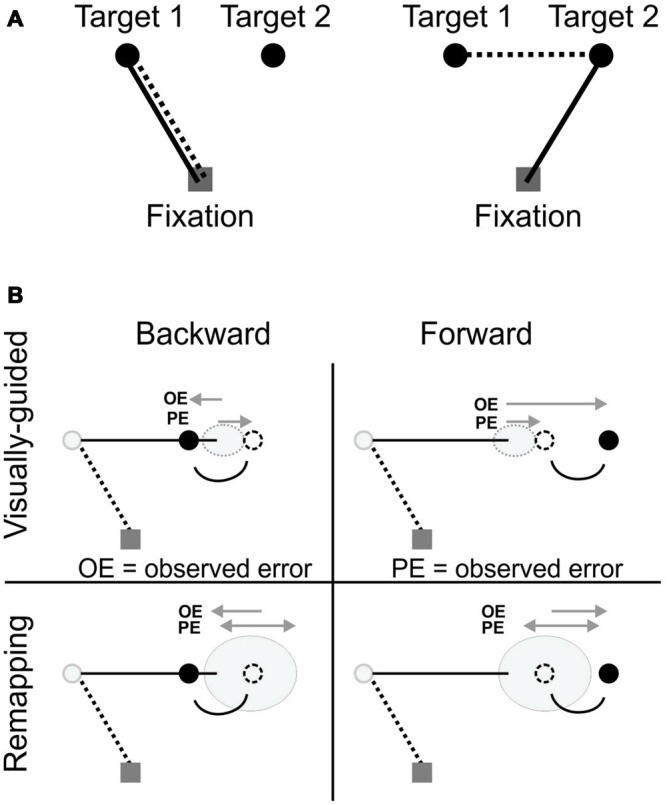
**(A)** The double-step task. While the participant fixates (gray square), targets 1 and 2 (black circles) are presented briefly. The participant’s task is to make a sequence of two saccades to the two targets. For the saccade to the target 1, the visual (solid line) vector matches the saccade vector (dotted line). However, after the 1st saccade, the visual vector to target 2 (solid line) no longer matches the saccade vector (dotted line). **(B)** Predictions. Predictions of the different error signals that could occur in the backward/forward sequential visually-guided and remapping conditions. OE, observed error; PE, predicted error; The black arrows represent the final eye position for the second saccade of a sequence. Gray circles represent the variability for the final eye position.

However, things are much more complicated in more complex environments, where only one of the multiple targets of interest can be fixated upon at a time. In this case, the retinal location of a potential visual target in the periphery may be held in memory during the execution of a first saccade to another target before being fixated upon. In this instance, the initial retinal vector and the final motor vector needed to capture the target do not match. One of the mechanisms involved in maintaining visual stability and motor accuracy across imperfectly planned and executed series of saccades is visual remapping: the retinal location of the target is held in short-term memory and its location is updated with each saccade (Wurtz, 2008). In visual stability, even if the retinal location of a target changes at each new fixation, it is still considered to be the same target. Remapping processes have been studied using the double-step saccade task (Hallett and Lightstone, 1976). Participants are asked to perform a sequence of two saccades toward the locations of two targets presented in brief succession (Figure 1A). The saccade to the first target can be guided based on the retinal vector of T1 since the visual (V1) and motor (M1) vectors align. However, for the second saccade, the motor vector (M2’) no longer matches with the visual vector (V2). In order to correctly plan this second saccade, the motor vector of the first executed saccade has to be taken into account (remapping process). Therefore, if at the end of the sequence of two saccades, an error occurs (e.g., because of a backward or forward target jump), and the actual error (i.e., observed error) is not equal to the predicted error, does the system attribute this to an error in the first and/or second saccade planning process or to imperfect remapping processes? Answering this question would provide more insight on how the brain maintains the accuracy of more complex saccades (i.e., remapped saccades), usually utilized in a more ecological environment, despite the occurrence of errors. To our knowledge, no study has investigated such aspects so far. To this aim, we propose to compare adaptation in two versions of the double-step paradigm. The first is a remapping task—where both targets are only presented before the onset of the 1st saccade. The second is a sequential visually-guided task—where the 2nd target was presented again at 1st saccade offset, allowing the second saccade to be visually-guided and thus not requiring any remapping of the 2nd saccade vector. If the system attributes the error to imperfect remapping processes rather than saccade planning processes, then adaptation may not occur at all in the remapping task.

Three conditions were tested for both the sequential visually-guided and remapping tasks: (1) adaptation to a forward target jump; (2) adaptation to a backward target jump; and (3) re-appearance of the target without any jump (control condition). We predicted that the greatest amount of adaptation would occur in the sequential visually-guided backward condition (Figure 1B), because the predicted error and the observed error would be highly different, i.e., in different directions. In contrast, for the sequential visually-guided forward condition, we expected smaller adaptation because the predicted and observed errors are in the same direction. This is because the undershoot produced by the forward target jump is already present, so therefore there is less drive to diminish this discrepancy. This is consistent with previous studies showing smaller adaptation for forward conditions (Straube et al., 1997; Noto et al., 1999; Panouillères et al., 2009). If remapped saccades do adapt, we predict similar amounts of adaptation in backward and forward conditions, as explained by Figure 1B. In the remapping task, the predicted error at the end of the 2nd saccade cannot be directly calculated from a peripherally viewed target, and we postulate that it is predicted from a remapped target location combining the initial retinal location of T2 (V2) and the efference copy of the first saccade (M1). The target location estimation would thus not only be inaccurate but also not necessarily under-estimated, i.e., the variability ellipse would be approximately centered on target location in remapping conditions. It would result in as many overshoots as undershoots, hence the predicted error would not be consistently in the same direction. Consequently the backward condition would not lead to greater adaptation than the forward condition.

## Materials and Methods

### Participants

Eight participants took part in the first study (three male, age range: 27–38, *M* = 32.25, including three authors: AK, DL and LP). All participants had normal vision and no known neurological impairments. Participants signed a consent form prior to the study. All procedures were approved by the local ethical committee on human experimentation (Comité de Protection des Personnes Sud-Est III), in agreement with French law (March 4, 2002) and the Declaration of Helsinki (number 2008-057B).

### Apparatus

**Figure 2:**
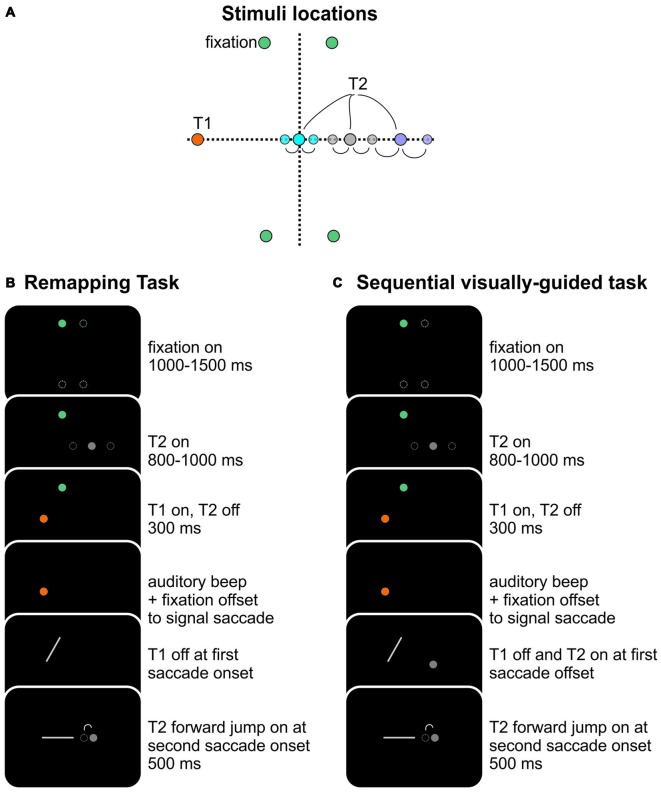
**Task stimuli and sequence. (A)** Stimulus locations for all targets are shown relative to screen center, which was located straight ahead of the two eyes. There were four possible fixation locations, shown in green, located 2.5° left and right and 10° up and down of center. T1, shown in orange, was 10° left and aligned horizontally with center. There were three possible T2 locations, aligned horizontally at 0° (blue), 5°(gray) and 10° (purple). The lighter filled dots show the backward and forward jump positions of the targets. The backward jump position for the 15° T2 target was the same as the forward jump position for the 10° T2 position (light gray dot right of solid gray dot). The backward and forward jumps were 15% of the T1-T2 amplitude. **(B)** Typical trial sequence for an adaptation trial. Each trial began with a fixation dot (solid green dot—one of four possible positions shown by the open dotted green circles, randomly selected). The fixation dot remained illuminated for a random period between 1000–1500 ms. Next, T2 was illuminated (randomly selected from three possible positions shown, and respectively located at 0°, 5°, and 10° right of center, in this case the 5° target). T2 remained illuminated for a random period between 800 and 1000 ms after which it was extinguished. At the same time T1 was illuminated (orange dot). After 300 ms, an auditory beep was sounded and the fixation dot was extinguished, signaling the participant to make a sequence of two saccades first to T1 then to T2. T1 was extinguished when the first saccade was detected (gray line). T2 reappeared at its new location at the second saccade onset. The example in the figure shows the backward jump position of the T2 target. **(C)** Sequential visually-guided task. The task sequence was identical to **(B)** except that T2 was re-illuminated at 1st saccade offset.

Participants sat in a completely dark room facing a vertical panel, whose center was aligned horizontally with the participant’s mid-sagittal plane and vertically aligned at eye level. The vertical panel was at a distance of 110 cm from the participant’s eyes. Red light emitting diodes (LED) were mounted onto the panel as shown in Figure 2A. There were four fixation LEDs (shown in green) located 10° above or below the center, at 2.5° left or right of center. One LED, aligned horizontally with the center and at 10° left, was the target for the first saccade (T1, orange circle). Three LEDs, also aligned horizontally with the center at a distance of 0° (blue circle), 5° (gray circle), and 10° (purple circle) right, were the targets for the second saccade (T2) and corresponded to target eccentricities of 10°, 15° and 20° from T1. Additional LEDs were located at 1.5° left, 2.75°, and 7.25° right, served as the targets for the backward jump during adaptation, corresponding to a jump of 15% of the target amplitude from T1 and T2. Two more LEDs located at 1.5° and 12.75° served as the forward jump targets for the 0° and the 10° T2 targets respectively. The 7.25° LED also served as the forward jump target for the 5° T2 target.

All targets were red LEDs (diameter: 3 mm) and were supplied by current levels which ensured illumination of the LED in darkness without any diffuse light. Custom-made software was used to illuminate the LEDs according to the sequence as described below.

The position of the left eye was recorded at 1000 Hz using an Eyelink 1000 video-based eye tracker (SR Research, Mississauga, ON, Canada). We recorded from the left eye as some participants could see the infrared light (wavelength of eye illumination, 910 nm) from the eye-tracker just below their eye. When using the right eye, the visible infrared light could be used as a reference for target positions, but this was not the case for the left eye as the infrared light was distant from the target array (all targets were located on the right side of the panel—see Figure 2B). Participants’ heads were stabilized using a tower mount system (SR Research) which supported the participants’ chin and forehead. Prior to each experimental block (except the post-test block), eye position was calibrated using a 9-point calibration window.

### Procedure

Participants took part in three sessions for the remapping task and three sessions for the sequential visually-guided task. For the remapping task, participants made a sequence of two saccades, where the position of T2 had to be remapped after the saccade to T1. For the sequential visually-guided task, T2 was re-illuminated after the saccade to T1, thus requiring no remapping. Each task (remapping and sequential visually-guided) was tested in three different conditions of target re-appearance after the second saccade: two with and one without a target jump (backward, forward and no-adaptation conditions, respectively), consisting of six different sessions. The six sessions were performed in random order for each participant, with at least 1 week apart to ensure no retention of adaptation (Alahyane and Pélisson, 2005).

A basic adaptation remapping trial is shown in Figure 2B. Each trial began with the illumination of one of the four fixation LEDs (green circles, randomly selected) on which participants were asked to fixate. After 1000–1500 ms, one of the three T2 targets (gray circles) was illuminated for 800–1000 ms. Next, it was extinguished and T1 was illuminated at the same time. After 300 ms, the fixation target was extinguished and a beep sounded. This was the signal for the participant to make a sequence of two saccades, first to T1 and then to the remembered location of T2 (as in Quaia et al., 2010). T1 was extinguished at the beginning of the first saccade. In the adaptation conditions, T2 reappeared at the forward (in this illustrated example) or backward jump location at the second saccade onset for 500 ms. In the no-adaptation condition, T2 reappeared instead at its initial location (no jump). After an inter-trial interval of 1 s, the next trial began.

Each session comprised four separate blocks. The first was a practice block, where T2 remained illuminated. The aim of the practice block was two-fold, first to allow participants to become familiar with the sequence of targets and saccades and second, to aid in resetting the gain in case there was any retention from a previous session even after a week. There were 48 trials in total (4 repetitions* 3 T2 targets* 4 fixations). Next there was a pre-test block, consisting of 36 trials (3 repetitions*3 T2 targets* 4 fixations). The pre-test sequence was identical to the basic practice trials described above except that target T2 was not re-illuminated after the 2nd saccade, and so participant was not provided with any feedback about the landing position of the 2nd saccade. Following the pre-test was an adaptation block, consisting of 240 trials. The trials were identical to the practice trial described above, where T2 either: (1) jumped back 15% of the amplitude from T1 to T2 at the beginning of the 2nd saccade (backward condition); (2) jumped forward 15% (forward condition); or (3) was re-illuminated at the same position (no-adaptation condition), for 500 ms at the second saccade onset for all conditions. The last block was a post-test, which was identical to the pre-test block.

The trial sequence and blocks were identical for the sequential visually-guided task. The main difference was that T2 was always re-illuminated at the first saccade offset and then was jumped (or not) at the second saccade onset (Figure 2C).

### Reduced Positions Control Experiment

In order to test further where the attribution of the error occurs (see “Introduction” Section, referring to the attribution of the error either on the T1 or T2 saccade target), we performed an additional control experiment for the remapping and the sequential visually-guided tasks with a reduced number of positions. Indeed, the lack of adaptation of the first saccade in the first experiment could be due to the variability of the first saccade direction due to the use of four different starting positions. Multiple fixation and T2 target locations could obscure the potential adaptation effect on T1. Therefore, we modified the protocol and kept only two (instead of four) fixations positions (right upper and lower green dots in Figure 2A) and only one (instead of three) T2 target positions (blue T2 position in Figure 2A). Participants performed only the adaptation blocks (both backward and forward). The methodology (e.g., participants, number of trials) remained the same as in the main experiment.

### Data Analysis

We collected a total of 17,280 trials from the eight participants (360 trials/session * 6 sessions each). During the experiments, the first and second saccades for each trial were detected automatically in real time using custom Matlab software, using a velocity threshold of at least 20°/s. This velocity threshold was adjusted between participants according to individual eye noise levels to keep the threshold as low as possible without artifactual triggering related to noise For offline data analysis, all saccades were detected using a velocity threshold of 50°/s and eye position was automatically extracted 50 ms before saccade onset and 50 ms after saccade offset. These points were also verified visually by the experimenter, thus ensuring that the eye position was only extracted during periods of stable fixation before and after each saccade. We removed trials where the participant executed the 1st saccade in anticipation (before the beep) or too late (more than 500 ms after), where the participant blinked during the saccades, and trials where there was noise in the signal. In total, 6.6% of trials were eliminated. In addition, we removed all trials with inaccurate fixations (0.6%).

To measure the influence of adaptation on the 1st saccade in the sequence, we investigated the horizontal endpoints of the 1st saccade. This was because the T2 jump was mostly perpendicular to the 1st saccade direction, thus if there was an influence of T2 jump on the 1st saccade, it would be in the horizontal direction and would be observable as a change in the horizontal endpoint (*x*-position) from the constant T1 position (10° left). We removed all trials with extreme 1st saccade endpoints, i.e., outside of a 10° horizontal and 5° vertical window of T1 (0.6%).

For the 2nd saccade in the sequence, we calculated the saccadic gain, calculated as the actual saccade amplitude divided by the desired saccade amplitude, which allowed us to collapse all different amplitudes for the different T2 targets into one comparable value. The actual saccade amplitude was the difference between the horizontal start and end positions of the 2nd saccade. The desired saccade amplitude was the difference between the horizontal start position of the 2nd saccade and the T2 target position (0°, 5° or 10°). Thus a gain of one would mean that the executed saccade accurately reached T2, whereas a gain less than one or greater than one would indicate, respectively, a hypometric or hypermetric executed saccade. To minimize the effect of outliers, we removed trials where the saccade gain was outside the mean gain ±2 SD for each block and subject (4.26% of all trials). This resulted in 15,201 remaining trials (88%).

Statistical analyses performed were repeated-measures analysis of variances (ANOVAs) and *t*-tests across the eight participants.

## Results

**Figure 3:**
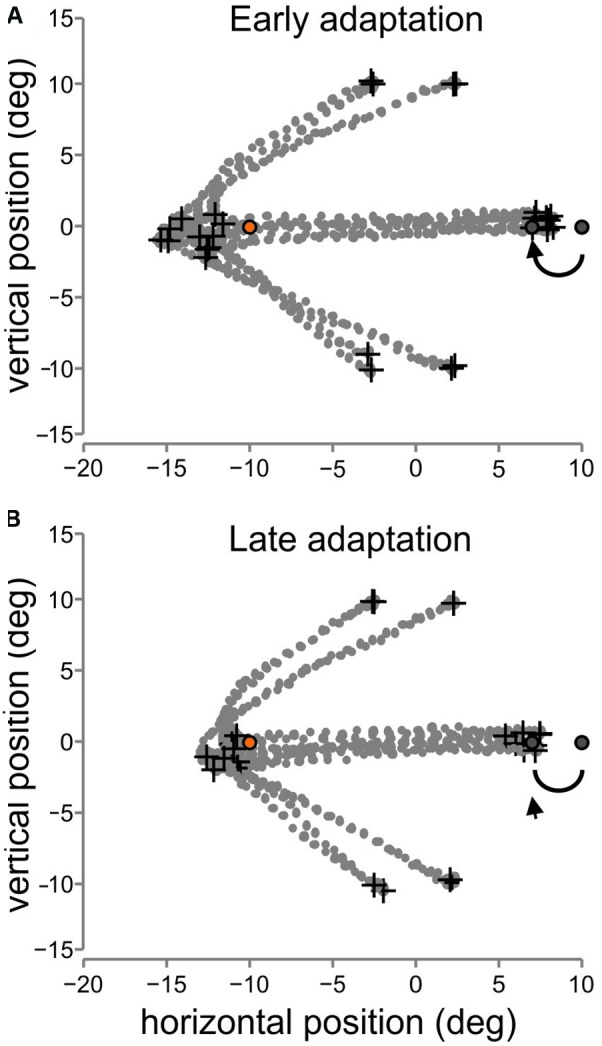
**Raw eye movement traces. (A)** Eye position traces are shown for eight trials early in adaptation (two per fixation position, black crosses demarcating fixation locations). The traces are from participant one for the backward adaptation block toward the 10° T2 position early in the block (within the first 40 trials). The traces show the two saccades from each fixation position to T1 (orange circle) and then from T1 to T2 (gray circles show the first T2 as well as the backward T2 jump position). The eye movement traces are shown in degrees relative to the center of the screen. **(B)** Eye position traces late in adaptation (last 60 trials, due to some signal loss).

We first confirmed that the adaptation paradigm was successful in inducing a change in saccade amplitude. For example, Figure 3 shows eye movement traces early (A) and late (B) in adaptation during the sequential visually-guided backward adaptation task for one participant, for the four fixation positions. In early adaptation (A), the participant made hypometric 2nd saccades from T1 (orange circle) to T2 (right-most gray circle) and then made a backward corrective saccade (not shown in the figure) in response to the T2 backward jump (black arrow and 2nd, left gray circle). Late in adaptation (B), the saccades from T1 to T2 are smaller, showing the effect of adaptation.

**Figure 4:**
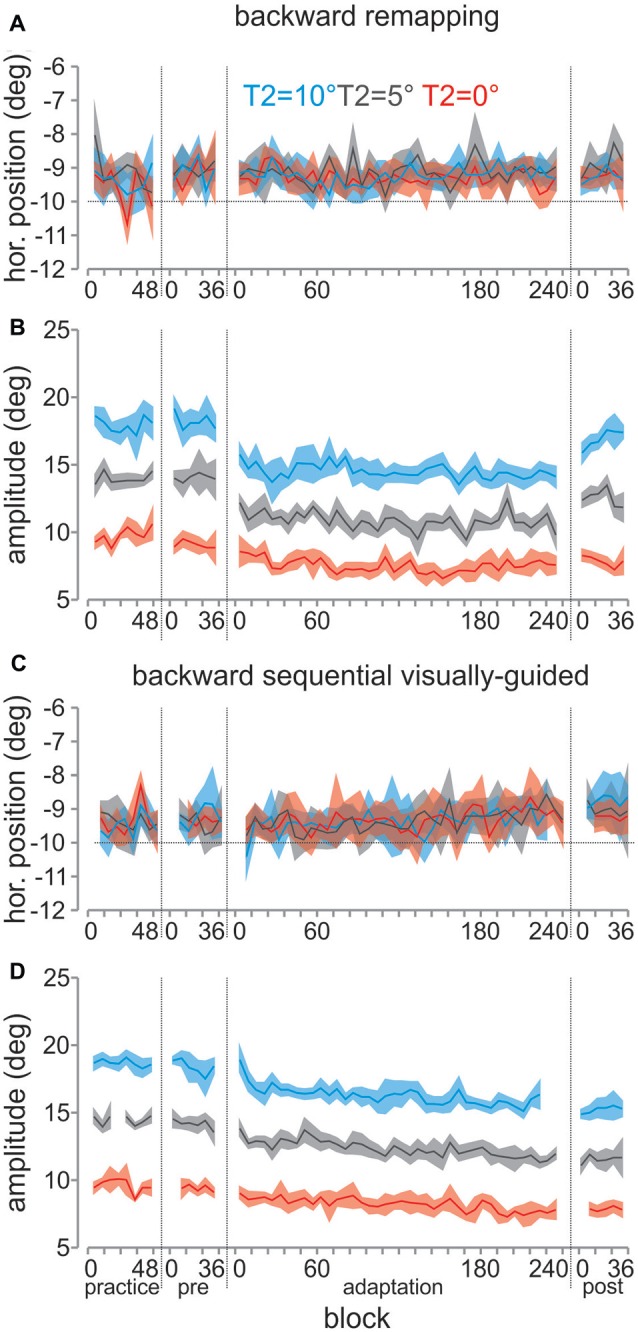
**Mean saccade horizontal endpoints and amplitudes for the backward conditions, across all participants. (A)** The horizontal endpoint of the 1st saccade from fixation to T1 is plotted over trial number (binned across 6 trials) in the order of blocks as they were presented for the backward remapping adaptation task. The three different colors correspond to the three T2 positions. Shaded colors represent SEM across all participants. The vertical dotted lines separate the different blocks. Trial number is shown in the *x*-axis within each block. **(B)** Amplitudes for the 2nd saccade from T1 to T2 are plotted over trial number in the same manner, for the backward remapping task. **(C)** Horizontal endpoints for the 1st saccade for the backward sequential visually guided task plotted in the same manner as **(A)**. **(D)** Amplitudes for the 2nd saccade for the backward sequential visually-guided task plotted in the same manner as **(C)**.

**Figure 5:**
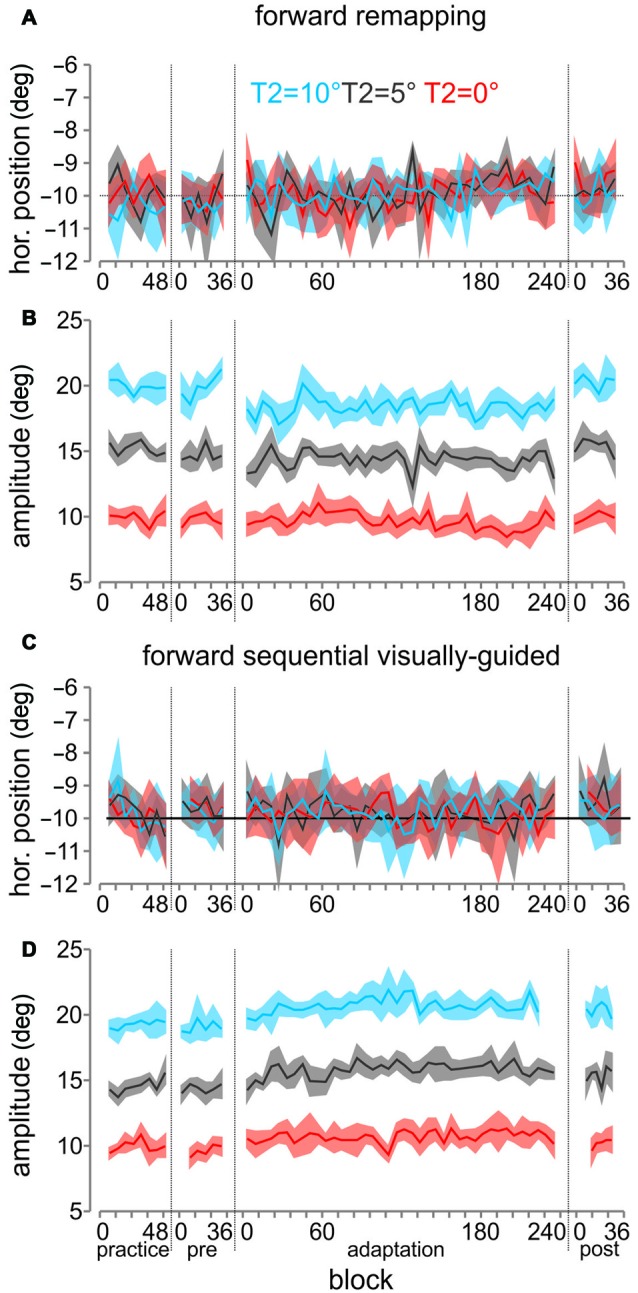
**Saccade horizontal endpoints and amplitudes for the forward conditions, across all participants. (A–D)** All panels are plotted in the same manner as for Figure 4.

We plotted the average horizontal endpoints (for the 1st saccade) and amplitudes (for the 2nd saccade) across all participants across all blocks comparing the sequential visually-guided and remapping tasks for backward (Figures 4A–D) and forward (Figures 5A–D) conditions. The average horizontal endpoints (six trial bins with SEM wings) of the saccades from fixation to T1 (A and C) and the amplitudes from T1 to T2 (B and D) are shown for the entire session, with separations indicating successive blocks of trials. The three different colors correspond to the different T2 positions. As can be seen in Figures 4A,C, 5A,C, the first saccade horizontal position was maintained throughout adaptation and into the post-adaptation block, with very little change. For the 2nd saccade (Figures 4B,D, 5B,D), during the adaptation block, saccade amplitudes for all three locations of T2 gradually increased or decreased, resulting in larger or smaller amplitude saccades during the post-adaptation block.

### Change of Horizontal Endpoint of the 1st Saccade

**Figure 6:**
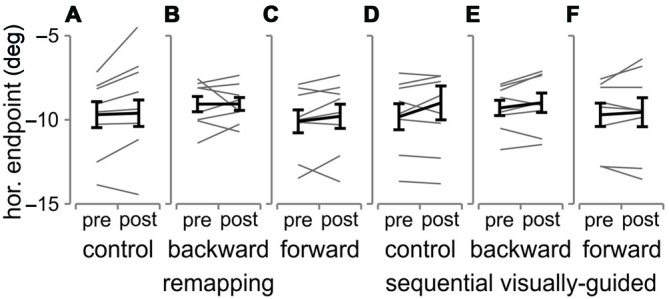
**Pre- and post-adaptation horizontal endpoints for the 1st saccade in the remapping and sequential visually-guided tasks**. Pre- and post-adaptation mean horizontal endpoints across all subjects (thick black line) and for each participant (thin gray lines) are shown for the three remapping **(A–C)** and the three sequential visually-guided **(D–F)** tasks. Error bars are SEM across all eight participants.

**Figure 7:**
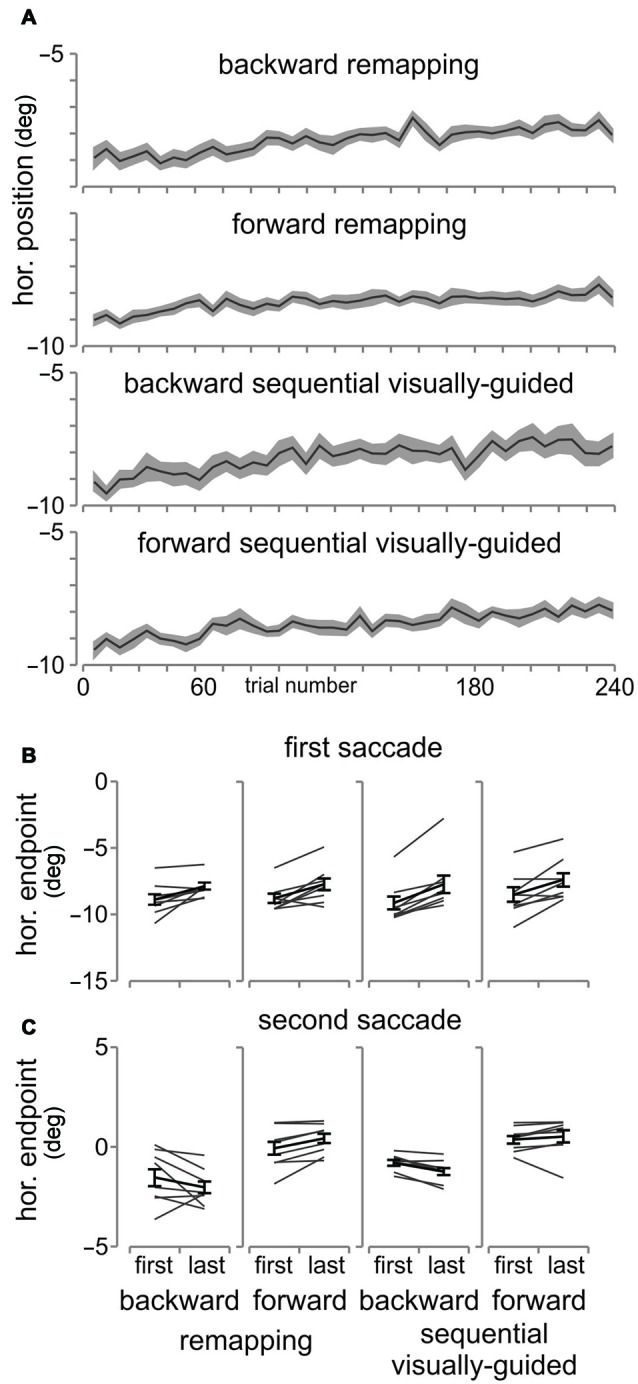
**Reduced position control experiment. (A)** Average 1st saccade endpoints. The 1st saccade endpoints are shown across all participants binned into six trial bins for all four conditions. The transparent wings are SEM **(B)** The average horizontal endpoints for the 1st saccade are shown for early (first 40 trials) and late (last 40 trials) across all participants and for each participant (gray lines). **(C)** Average horizontal endpoints for the 2nd saccade shown in the same manner as **(B)**.

We investigated whether adaptation led to changes in the horizontal endpoints of the 1st saccade to T1 for both tasks in all three conditions. During the remapping adaptation task, participants were asked to make two saccades in a sequence and only received feedback of the position of T2 (jumped backward, forward or at the same location) after the 2nd saccade (Figure 2B). Thus the saccadic system has no way of knowing whether it was the 1st or 2nd saccade that was erroneous and required correction and so it may modify the direction, reflected in the horizontal endpoint, of the 1st saccade from fixation to T1. We tested whether this was the case. In Figure 6 are plotted the mean horizontal endpoints of the 1st saccade (see “Materials and Methods” Section) for the pre- and post-blocks across all participants, and shown for each of the three conditions in each of the two tasks. We performed repeated measures *t*-tests comparing pre- and post-horizontal endpoints for each condition (backward, forward or no adaptation) separately for the remapping and sequential visually-guided tasks. For the remapping task (A–C), there was no systematic change in the endpoint of the 1st saccade for any of the three conditions (all 3–*t*_(7)_ < 1, *p* > 0.05). This was also the case for the sequential visually-guided (D–F) task (all 3–*t*_(7)_ < 2.3, *p* > 0.05), but this result was expected since T2 was re-illuminated after the first saccade to T1 and so the saccade to T2 was visually-guided. We confirmed this same result for the reduced positions control experiment. In Figure 7A are plotted the average horizontal endpoints for the 1st saccade across the four conditions/tasks and in Figure 7B, the mean horizontal endpoints for T1 for the first 40 and last 40 adaptation trials for all four conditions/tasks along with individual participant data also shown (gray lines). As can be seen, we observed a rightward shift in the first saccade endpoints, but this was the case for all conditions, regardless of whether the target jump was backward or forward. Therefore this rightward shift cannot be considered as adaptation of the first saccade. In contrast, there was a consistent change in the 2nd saccade endpoints depending on the target jump direction (Figure 7C). Taken together, we found no evidence of adaptation of the 1st saccade in the remapping task (nor in the sequential visually-guided task).

### Gain Changes for the 2nd Saccade in The Remapping Task

Next we investigated whether there were changes in gain for the 2nd saccade from the adaptation for the remapping as well as the sequential visually-guided tasks. We used gain for analyses and figures because we could then collapse across the three T2 locations and because this allows easier comparison across conditions, tasks and other studies. As above, we compared pre- and post-gains for the 2nd saccade for each condition and task.

**Figure 8:**
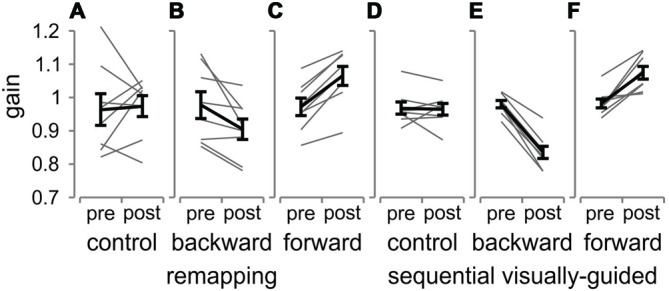
**Pre- and post-adaptation mean gains for the 2nd saccade in the remapping and sequential visually-guided tasks**. Pre- and post- adaptation mean gains are shown for each participant (thin gray lines) and pooled across all participants (thick black lines) for the control, backward and forward conditions for the remapping **(A–C)** and the sequential visually-guided **(D–F)** tasks. Error bars are SEM across all trials within the block.

In Figures 8A–C are shown the pre- and post-adaptation mean gains across all participants (and individual participants as gray lines) in the control (A), backward (B) and forward (C) remapping conditions. We performed a two-way ANOVA with the three conditions (backward, forward and control) and the pre- and post-blocks as factors, which revealed no significant main effect for block (*p* > 0.05), a significant main effect of condition (*F*_(2,14)_ = 8.63, *p* = 0.004) and a significant interaction effect (*F*_(2,14)_ = 10.16, *p* = 0.002). These findings suggest that there was a change in gain from pre- to post-blocks that was different for the different conditions. As can be seen in the figure, the change in gain in the control condition was very variable for the remapping task (A) compared to the sequential visually-guided task (D) but there was no consistent pattern of gain difference between the pre- and post-adaptation blocks (*t*_(7)_ = 0.3, *p* > 0.05). In contrast, for the backward condition (B), there was a decrease in gain (mean decrease of 0.07, *t*_(7)_ = 2.717, *p* < 0.05). For the forward adaptation condition (C), all participants showed consistent increases in gain, resulting in a significant effect at the group level (*t*_(7)_ = 6.95, *p* < 0.001). Table 1 lists the mean amplitudes for the pre- and post-blocks for each T2 position for all three conditions.

**Table 1:** Mean amplitudes in deg (*SD across participants) for each T2 position for each pre- and post-block for all conditions.

Condition	T2 in degree	Pre-block mean amplitude in degree (SD)	Post-block mean amplitude in degree (SD)	Difference in amplitude
Control remapping	0°	9.03 (2.24)	9.10 (2.9)	0.07
	5°	13.87 (2.83)	13.83 (3.4)	−0.04
	10°	18.98 (4.05)	18.29 (3.71)	−0.69
Backward remapping	0°	9.10 (1.98)	7.76 (1.9)	−1.35
	5°	14.06 (2.81)	12.53 (2.4)	−1.53
	10°	18.02 (3.75)	17.06 (2.54)	−0.96
Forward remapping	0°	9.69 (2.46)	9.95 (2.78)	0.25
	5°	14.45 (3.06)	15.43 (3.02)	0.97
	10°	19.92 (3.5)	20.21 (3.13)	0.29
Control sequential	0°	9.77 (3.04)	8.80 (3.48)	−0.97
	5°	14.41 (2.81)	13.51 (3.54)	−0.90
	10°	18.99 (3.3)	18.08 (3.4)	−0.90
Backward sequential	0°	9.38 (1.75)	7.83 (2.03)	−1.56
	5°	14.23 (1.9)	11.56 (2.02)	−2.67
	10°	18.51 (2.24)	15.11 (2.23)	−3.40
Forward sequential	0°	9.67 (2.69)	10.24 (3.27)	0.57
	5°	14.36 (2.27)	15.39 (3.07)	1.03
	10°	18.88 (2.7)	20.31 (3.18)	1.43

In sum, participants also showed significant changes in gain between pre- and post-blocks for the remapping tasks across the different target jump conditions. These results show that remapped saccades are adapted and that the adaptation takes place entirely on the 2nd saccade in the sequence. However, the amount of adaptation differed slightly between the sequential visually-guided and the remapping tasks, as detailed below.

### Gain Changes for 2nd Saccade in The Sequential Visually-Guided Saccade Task

We also tested the effects of adaptation on the 2nd saccade for the sequential visually-guided task. Figures 8D–F shows the mean gain for the pre- and the post-blocks across all eight participants for the control, backward and forward adaptation conditions.

A two-way ANOVA was performed for the sequential visually-guided saccade tasks with the three conditions (backward, forward and control) and the pre- and post-blocks as factors. As in the remapping condition, there was no significant main effect for block (*p* > 0.05), a significant main effect of condition (*F*_(2,14)_ = 102.49, *p* = 0.000) and a significant interaction effect (*F*_(2,14)_ = 68.1, *p* = 0.002), suggesting a different change in gain for the different conditions. As can be seen, in the control condition (D), where the T2 target did not jump during the 2nd saccade from T1 to T2, there was no consistent change in gain from the pre- to the post-adaptation condition; some participants showed a slight decrease, some a slight increase and some no change at all. *Post hoc* paired *t*-tests showed no significant difference of mean gain between the two blocks (*t*_(7)_ = 0.2, *p* > 0.05). In contrast, for the backward adaptation condition (E), participants showed a decrease in gain that was significant at the group level (*t*_(7)_ = 12.5, *p* < 0.001). Likewise, for the forward adaptation (F), there was a significant increase in gain (*t*_(7)_ = 4.824, *p* < 0.01), as observed in all participants although to a reduced extent in participants 3 and 7. Table 1 lists the mean amplitudes for the pre- and post-blocks for each T2 position for all three conditions. In summary, for the sequential visually-guided task, participants showed significant changes in gain in the 2nd saccade in response to both a backward and a forward step of T2.

### Comparing Adaptation Between Remapped and Sequential Visually-Guided Tasks

**Figure 9:**
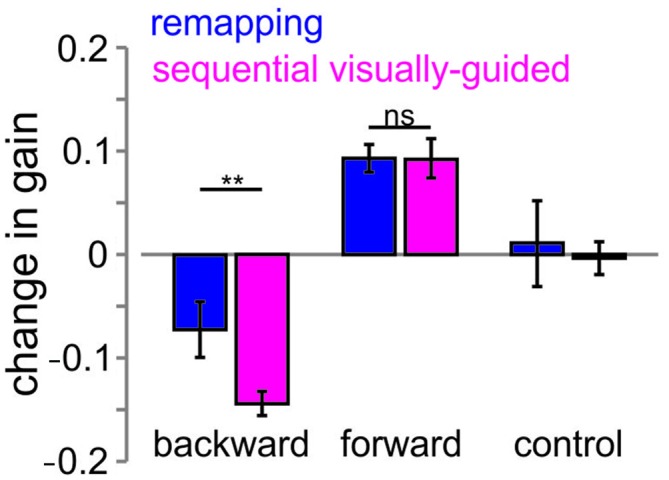
**Change in gain across the remapping and sequential visually-guided saccade tasks**. The change in gain from pre to the post adaptation block is plotted for the remapping (blue bars) and the sequential visually-guided (pink) tasks and for the backward, forward and control conditions. Error bars are SEM across participants. Ns = non-significant difference. **p < 0.01.

To compare the saccadic gain change in the 2nd saccade between the remapped and sequential visually-guided saccades, we plot in Figure 9, the mean change (pre-adaptation vs. post-adaptation) computed over all subjects for the backward, forward and control jump conditions. The change in gain was calculated for each participant and the mean value across participants is shown.

We compared the change in gain for the backward and forward adaptation conditions for the remapping (blue) and sequential visually-guided (pink) tasks using a two-way repeated measures ANOVA with task and condition as factors. We performed this analysis on the absolute gain change to compare the magnitude (rather than the direction) of the gain change. This analysis resulted in a significant main effect for task (*F*_(1,7)_ = 6.8, *p* < 0.05) and a significant interaction effect (*F*_(1,7)_ = 6.4, *p* < 0.05) but no effect for condition (*p* > 0.05). As can be seen, there was a significant difference in the gain decrease in the backward target jump condition (right two bars) for both tasks. The decrease in gain for the remapping task was 0.075, which was almost half of that for the sequential visually-guided task (0.144; Holm-Bonferroni *post hoc* corrected—*t*_(7)_ = 3, *p* < 0.02). This translated to a decrease of 50% of the target jump for the remapping task as compared to an almost complete adaptation for the sequential visually-guided task (0.14–96%). In contrast, for the forward condition, the change in gain was similar between the remapping and sequential visually-guided tasks (rightmost two bars, 0.093 and 0.092 respectively, *p* > 0.05). This translated to approximately 62% of the target jump in the remapping task and 61% in the sequential visually-guided task.

We confirmed that there were no significant differences in pre-adaptation gain between the remapping and the sequential visually-guided tasks in any of the target jump conditions (*p* > 0.05); thus these differences in the change in gain were not due to differences in gain in the pre-adaptation condition.

In summary, whereas the change in gain of the 2nd saccade was similar for both the remapping and the sequential visually-guided tasks for the forward target jump condition, it was much less in the remapping task than the sequential visually-guided task for the backward condition.

### Comparing Saccadic Parameters (Latency, Variability and Gain) Between Remapped and Sequential Visually-Guided Saccades

In the sequential visually-guided saccade task, the 2nd target was re-illuminated at the end of the 1st saccade, which could be used to trigger the 2nd saccade. In the remapping task, the target was not re-illuminated, and so the 2nd target was not triggered by a visual onset. Nevertheless, a two-way repeated measures ANOVA showed no main effect for task (*p* > 0.05) and no interaction effect between session and task for the latencies of the second saccades (*p* > 0.05). We found that on average the inter-saccadic time interval for the remapping task was 402, 424 and 414 ms (pre-, adapt and post-sessions) and for the sequential visually-guided task was 410, 466 and 429 ms. We also investigated the differences in variability and mean gain between remapped and sequential visually-guided second saccades during the pre-adaptation block (Table 2). Standard deviations (SDs) for the 2nd saccade gain during remapped saccades were significantly higher than during sequential visually-guided saccades across all three conditions (two-way ANOVA with task and condition as factors; task *F*_(1,7)_ = 34, *p* < 0.01; condition, *p* > 0.05; interaction, *p* > 0.05). In contrast, there was no difference in the SD for the horizontal endpoints for the 1st saccade in any condition (*p* > 0.05).

**Table 2:** **Baseline saccadic parameters measured during the pre-test for the 2nd saccade**.

Condition	SD gain	Mean gain	Percentage of undershoots
Backward remapping	0.13	0.98	57%
Forward remapping	0.12	0.97	58%
Backward sequential	0.05	0.98	66%
Forward sequential	0.05	0.98	68%

Table 2 also shows that, as we predicted, the proportion of undershoots was similar for the backward and forward remapping conditions, with almost as many overshoots as undershoots (57% and 58% undershoots for backward and forward respectively). This was due to the large variability in saccade endpoints rather than a difference in mean gain (which was not different in the different tasks—Table 2). In contrast, in the sequential visually-guided task, there was a much smaller variability and thus a larger percentage of undershoots compared to overshoots.

## Discussion

To summarize our results, remapped saccades were significantly adapted in response to both a backward and a forward target shift. The target jump adapted the second saccade and not the first saccade in the sequence, suggesting that the error was attributed to the second saccade planning only. The fact that the second saccade can be adapted suggests that its planning involves the visuo-motor transformation of a horizontal visual remapped vector instead of a direct remapping of the motor vector associated with the initial visual presentation of target 2. The amount of adaptation was similar for both forward and backward target jumps, corresponding to about half the amount of the jump. This amount of adaptation was similar to that obtained in the forward sequential visually-guided saccade task, but not to in the backward sequential visually-guided saccade task, which showed an almost complete adaptation to the target jump.

### Sequence of Two Saccades with Remapping are Adaptable and Error is Attributed to the Second Saccade Only

We tested whether remapped saccades were adaptable and found that indeed, a systematic target jump backward or forward led to a significant amount of adaptation. In addition, as shown in the results, the adaptation induced by the target jump occurring at the end of the sequence only occurred on the second saccade but not on the first saccade of the sequence. We did observe a rightward shift of T1 for the adaptation conditions in the reduced position control experiment, but this did not depend on whether it was a forward or backward condition. These results confirm that the 1st target was not adapted in response to the T2 target jump, but rather that the shift in endpoint toward the T2 target location might be an implicit change to reduce the amplitude of the 2nd saccade. These two observations together suggest that: (1) the second saccade was planned after the execution of the first saccade rather than at the visual presentation of the two targets; and (2) the error was attributed to the second saccade planning (the visuo-motor transformation of the remapped visual vector) rather than to memory and remapping processes.

First, consistent with other studies (Bellebaum et al., 2005; Munuera et al., 2009), we show that remapping processes and the planning of the second saccade occur after the execution of the first saccade and the system takes the actual final eye position after the first saccade into account to compute the motor vector required to execute the saccade to the remapped target location. However, even if there are two sequential planning processes, since we did not provide visual feedback of the first saccade landing position, there were not two sequential error signals to observe. Presumably, because the first saccade was visually-guided, the system trusted its reliability and did not modify this first saccade.

Second, even if the actual first saccadic displacement is taken into account, the second saccade generally results in higher endpoint variability compared to visually-guided saccades, presumably due to noisy remapping processes after the first saccade execution. These remapping processes could occur at the visual level (goal updating hypothesis of Quaia et al., 2010), i.e., the combination of V2 and M1 to compute a remapped visual vector V2’ which is then transformed into a motor vector. Alternatively, the remapping processes could occur at the motor level (motor updating hypothesis of Quaia et al., 2010; see also Ethier et al., 2008), i.e., a motor remapped vector M2’ is directly computed from the subtraction of M1 and the motor vector resulting from the visuo-motor transformation of V2. The motor updating hypothesis would have predicted that the planning process of the second saccade after the first saccade execution would not involve a visuo-motor transformation and would thus not be adaptable at all (or only at a general motor level). Our results are therefore in favor of a remapping at the visual level, in line with functional magnetic resonance imaging (Medendorp et al., 2003; Merriam et al., 2003, 2007), neurophysiological (Colby and Goldberg, 1999; Nakamura and Colby, 2002) as well as neuropsychological studies (Khan et al., 2005a,b; Blangero et al., 2011) which have suggested that remapping of target locations across saccadic eye movements takes place at the level of the parietal cortex or before (in the occipital cortex).

### Backward vs. Forward Adaptation

As others have shown for simple visually-guided saccades, we also found that backward adaptation induces much stronger adaptive changes than forward adaptation for sequential visually-guided saccades (Straube et al., 1997; Noto et al., 1999; Panouillères et al., 2009). This consistent difference in the amount of adaptation has been considered to be evidence that separate mechanisms are involved in these two types (backward and forward) of adaptation (Ethier et al., 2008). Backward adaptation is believed to take place at the motor level and in the cerebellum (Frens and van Opstal, 1994; Melis and van Gisbergen, 1996; Wallman and Fuchs, 1998; Edelman and Goldberg, 2002; Hopp and Fuchs, 2002; Alahyane et al., 2004, 2007; Kojima et al., 2008), whereas forward adaptation would tap into higher saccadic system levels involving target encoding and/or visuo-motor transformation (Wallman and Fuchs, 1998; Zimmermann and Lappe, 2010, 2011). For remapped saccades, the brain presumably attributes the error to imperfect localization processes rather than to general motor execution, which could explain why—backward—“behaves” like a forward adaptation, with adaptation amount similar to forward sequential visually-guided adaptation. This would be the case also for memory-guided or anti-saccades (Fujita et al., 2002; Alahyane et al., 2007; Cotti et al., 2009; Lévy-Bencheton et al., 2013, 2014). Possible neural substrates for memory-guided saccades adaptation has been put forward based on a study of Parkinson’s disease (PD) patients, thus suggesting an involvement of the basal ganglia or of the output of the dorso-lateral prefrontal cortex (DLPFC) to basal ganglia (MacAskill et al., 2002).

Note that, the amount of adaptive gain change cannot be explained by only the greater amount of variability of the saccade endpoints in remapping conditions, since this should have also reduced the amount of gain change for forward target jumps. In contrast, we observed similar gain changes in forward sequential visually-guided and remapping conditions, even though the variability was greater for the remapped saccades. Also, the amount of gain change cannot be explained only by the mean gain of remapped saccades, which was not different from sequential visually-guided saccades in pre- condition. We suggest rather that our results emerge from the combination of these two parameters (see Figure 1B and Table 2), which leads to systematic undershoots in sequential visually-guided conditions (predicted error systematically in the direction of the saccade) but to as many undershoots as overshoots in remapped conditions (predicted error with no systematic direction). Therefore, the highest amount of adaptation in backward sequential visually-guided condition can be related to the systematically opposite directions of the observed errors (systematic overshoots) with respect to the predicted errors (systematic undershoots) during the adaptation exposure.

In conclusion, while remapped saccades can be adapted, the adaptation mechanisms, and possibly the underlying neural substrates, are likely different from those in backward sequential visually-guided saccades.

## Author Contributions

DL-B and AZK contributed equally to the article. Both designed the experiment, ran subjects, analyzed the data and wrote the article. DP was involved in designing the experiment, interpreting data and writing the article. CT was involved in intepreting data, writing and editing the article. LP was involved in designing the experiment, intepreting the data, writing and editing the article.

## Conflict of Interest Statement

The authors declare that the research was conducted in the absence of any commercial or financial relationships that could be construed as a potential conflict of interest.
